# Phase I single and multiple dose study to evaluate the safety, tolerability, and pharmacokinetics of BMS-927711 in healthy subjects

**DOI:** 10.1186/1129-2377-14-S1-P118

**Published:** 2013-02-21

**Authors:** G Tong, I Savant, N Jariwala, D Burt, N Zheng, A Buzescu, R Bertz, S Keswani, R Marcus

**Affiliations:** 1 Bristol-Myers Squibb, USA

## Introduction

Calcitonin gene-related peptide (CGRP) may play a causal role in migraine. Blocking the CGRP receptor may be an effective approach to migraine relief while avoiding the vasoconstrictive effects associated with triptans. BMS-927711 is a potent, selective, CGRP receptor antagonist. The

## Objective

of this Phase I study was to assess the safety, tolerability, and pharmacokinetics of single (SAD) and multiple ascending (MAD) oral doses of BMS-927711 in healthy subjects.

## Methods

For SAD, 8 healthy subjects received a single dose (25,75,150,300,600,900, or 1500 mg) of BMS-927711 or placebo. For MAD, 8 healthy subjects received a daily dose (75,150,300,450, and 600 mg) or 300 twice daily of BMS-927711 or placebo for 14 days.

## Results

BMS-927711 was well tolerated at single doses up to 1500 mg and at multiple doses up to 600 mg for 14 days. There were no serious adverse events (AEs). The maximum tolerated dose was not reached. The most common AEs among BMS-927711 groups during SAD were nausea (n=7 BMS; n=0 placebo, and dizziness (n=5 BMS; n=0 placebo), and were constipation (n=8 BMS; n=3 placebo) and headache (n=8 BMS; n=2 placebo) during MAD. Most AEs were mild in nature. 2 subjects discontinued the MAD due to skin rash, and 1 discontinued due to elevated creatinine. BMS-927711 antagonizes CGRP-induced increases in marmoset facial blood flow (a surrogate marker for intracranial artery dilation) with 75% inhibition at ~700 nM, a surrogate for efficacious exposure. Following single doses, the plasma exposures exhibited biphasic disposition with a terminal T1/2 of ~10-12 hrs. At 2 hours (SAD), ≥700 nM exposure was achieved in most subjects at doses ≥75 mg. Food delayed Tmax (from 1 to 4 hrs post dose), and famotidine coadministration reduced bioavailability (Cmax ~26%, AUC ~42%).

## Conclusions

BMS-927711 appeared to be safe and well tolerated over a range of doses in this healthy population. After single oral doses ≥75 mg, clinical exposures are above the efficacious margin of 700 nM at 2 hrs post dose, supporting further clinical development in acute migraine.

